# Brazilian version of the ACTIVLIM: translation, cultural adaptation, and validation for neuromuscular disorders

**DOI:** 10.1055/s-0045-1814398

**Published:** 2026-01-25

**Authors:** Ana Carolina Costa Santos, Daniela Melo de Almeida, Nathalia de Brito Pereira, André Macedo Serafim Silva, Edmar Zanoteli, Mariana Callil Voos

**Affiliations:** 1 Universidade de São Paulo, Faculdade de Medicina, Departamento de Neurologia, São Paulo SP, Brazil.; 2 Pontifícia Universidade Católica de São Paulo, Faculdade de Ciências Humanas e da Saúde, Curso de Fisioterapia, São Paulo SP, Brazil.

**Keywords:** Motor Activity, International Classification of Functioning, Disability and Health, Motor Neuron Disease, Neuromuscular Junction Diseases, Social Participation

## Abstract

**Background:**

The Activity Limitation Measure (ACTIVLIM) is a self-reported instrument consisting of 22 daily activity items graded on 3 levels (easy, difficult, or impossible).

**Objective:**

To translate, culturally adapt, and validate the Brazilian Portuguese version of the ACTIVLIM for individuals with neuromuscular disorders.

**Methods:**

The present was a cross-sectional observational study. The translation process followed standardized guidelines, including steps such as forward translation, synthesis, back-translation, expert committee review, and pretesting (psychometric analysis). A total of 268 individuals with neuromuscular disorders filled out the Brazilian ACTIVLIM. Test-retest reliability was assessed in a subgroup of 60 participants, who were evaluated twice by the same physiotherapist with an interval of one month.

**Results:**

The intraclass correlation coefficient (ICC) for intrarater reliability was of 0.95. Internal consistency was high (Cronbach's alpha = 0.940). External validity showed strong correlations involving ACTIVLIM scores and the scores on the Vignos scale (r = −0.907), the Brooke scale (r = −0.908), and the Functional Independence Measure (r = 0.864), all with *p* < 0.001. Proximal muscle strength in the upper (r = 0.748) and lower limbs (r = 0.793), measured through the Medical Research Council scale, also correlated significantly with ACTIVLIM scores. Linear regression identified that the scores on the Vignos (R ^2^ = 0.8236), and Brooke scales (R ^2^ = 0.8132), as well as proximal muscle strength in the lower (R ^2^ = 0.6480) and upper limbs (R ^2^ = 0.5805), were the main predictors of ACTIVLIM variance.

**Conclusion:**

The Brazilian Portuguese version of the ACTIVLIM demonstrated strong reliability and validity. Its scores were significantly associated with disability level, functional independence, and muscle strength in individuals with neuromuscular disorders.

## INTRODUCTION

Rehabilitation seeks to reduce vulnerability and improve the quality of life of individuals with disabilities by enhancing their daily performance. For an effective intervention, health professionals must have a clear understanding of each patient's abilities and limitations. Standardized protocols to assess health status and physical functioning are essential tools to monitor treatment outcomes and guide rehabilitation strategies. 1

Furthermore, the development of new pharmacological therapies has heightened the demand for precise and sensitive outcome measures. Participation, understood as the opportunity to exercise citizenship and autonomy—including concepts such as freedom, agency, and subjectivity—has become a central focus in rehabilitation. Addressing participation requires integrating the individual and collective dimensions of care, which can pose challenges in the clinical practice, particularly for physiotherapists. 2

Individuals with neuromuscular disorders (NMDs) commonly experience progressive muscle weakness, loss of functional independence, and significant impairments in daily activities. 3 4 Many are unable to perform basic tasks such as rising from a chair, climbing stairs, or taking a shower. 5 6 These limitations in self-care often result in reduced social participation.

The Activity Limitation Measure (ACTIVLIM), developed and validated by Vandelverde et al., 7 is a self-reported instrument designed to assess perceived limitations in daily self-care activities. Based on the concept of activity limitation as defined by the International Classification of Functioning, Disability and Health (ICF), the ACTIVLIM is a unidimensional questionnaire that addresses domains such as mobility, self-care, and domestic activities. It evaluates basic body movements associated with tasks in which individuals with NMDs may require assistance or support. 8

Although the ACTIVLIM addresses highly-relevant aspects of functioning—particularly independence in self-care and mobility—its use in different cultural and linguistic contexts requires rigorous adaptation. The cross-cultural adaptation of clinical assessment tools must follow a systematic process to preserve the instrument's content, psychometric properties, and validity. Ensuring semantic and psychometric equivalence in the translated version is essential for its reliability and clinical usefulness. 9 10

Therefore, the aim of the present study was to translate, cross-culturally adapt, and validate the Brazilian Portuguese version of the ACTIVLIM, assessing its psychometric properties in individuals with NMDs.

## METHODS

The present cross-sectional observational study was approved by the Ethics in Research Committee of Hospital das Clínicas da Faculdade de Medicina da Universidade de São Paulo (HCFMUSP), under protocol number 3.264.694. Prior to initiating the translation and cross-cultural adaptation processes of the ACTIVLIM, authorization was obtained from the Physical Medicine and Rehabilitation Unit of Université Catholique de Louvain, the original developers of the scale.

### Translation and cross-cultural adaptation

The translation and adaptation processes followed the six standardized steps proposed by Beaton et al.: 9

1. Initial translation from English to Brazilian Portuguese by two independent translators, one of whom was a physiotherapist;
2. Synthesis of the two translations into a single version;
3. Back-translation of the synthesized version into English by two native English speakers blinded to the original version;
4. Expert committee review, including specialists in neuromuscular diseases, methodology, and linguistics;
5. Pretesting of the prefinal version to assess comprehensibility and clarity in a sample of 60 individuals with NMDs; and
6. Assessment of psychometric properties, including reliability and validity.

### Participants and inclusion criteria

The participants were recruited from the Neurology Clinic at HCFMUSP between August 2018 and July 2019. All individuals who met the inclusion criteria and agreed to participate signed an informed consent form. In total, 268 participants were included in the study. For minors, assents were obtained from the child, and informed consent was signed by their parents or legal guardians.

The eligible participants had confirmed diagnoses of NMDs, including spinal muscular atrophy (SMA), Steinert's myotonic dystrophy, limb-girdle muscular dystrophy, dystrophinopathies (Duchenne/Becker), congenital myopathies, hereditary facioscapulohumeral dystrophy, inflammatory myopathies, acquired neuropathies, amyotrophic lateral sclerosis (ALS), and investigational myopathies and myasthenias. All diagnoses were confirmed though genetic testing. Sociodemographic data such as age and level of schooling were collected at the time of evaluation ( Table 1 ).

**Table 1:** Characteristics of the study sample

		n (%)
**Gender**	Male	148 (55%)
	Female	120 (45%)
**Age (range: 6–78 years)**	Children (range: 6–15; Mean: 9.4 years)	25 (10%)
	Adults (range: 16–78; Mean: 42.4 years)	243 (90%)
Mean age		39.3 years
Schooling* (mean: 9.6 years)		Range: 4–16 years
**Disease stages**	Gait	226 (84%)
	Wheelchair	42 (16%)
**Diagnosis classification**	Spinal muscular atrophy	18 (7%)
	Steinert's myotonic dystrophy	32 (12%)
	Congenital dystrophy and/or myopathy	22 (8%)
	Girdle muscular dystrophy	37 (14%)
	Dystrophinopathies (DMD/DMB)	23 (9%)
	Fascioescapulohumeral	10 (4%)
	Inflammatory myopathy	16 (6%)
	Mitochondrial myopathy	33 (12%)
	Others**	77 (28%)
**Rating average**	ACTIVLIM	25.1
	Vignos scale	2.0
	Brooke scale	3.5
	MIF	102.2
**Standard deviation**	ACTIVLIM	8.6
	Vignos	1.2
	Brooke	2.0
	MIF	19.4

Abbreviations: ACTIVLIM, Activity Limitation Measure; BMD, Becker muscular dystrophy; DMD, Duchenne muscular dystrophy; FIM, Functional Independence Measure.

Notes: *In children, we considered the parents' education. **Neuropathies, myasthenia, amyotrophic lateral sclerosis, myopathies under investigation.

### Assessment

The assessment protocol was structured according to the conceptual framework of the ICF, which provides a comprehensive model to illustrate the relationships among health conditions, functioning, and contextual factors. 11

The ACTIVLIM, which can be applied to adults and children, comprises 22 items: 18 of them are common to all age groups, covering domains such as dressing, personal hygiene, fine motor skills, walking, and stair navigation; and 4 are age-specific items. For individuals aged 16 years and older, the additional items include carrying a heavy package, getting into a car, walking more than 1 kilometer, and standing for long periods. For children aged 6 to 15 years, age-appropriate items include closing a door, jumping on one foot, putting on a backpack, and running. 7

With this structure, the ACTIVLIM can be used across the lifespan, enabling longitudinal tracking of functional changes in individuals with NMDs. It facilitates comparisons among patients of different age groups with the same diagnosis, thus capturing the clinical heterogeneity of myopathies, dystrophies, neuropathies, and motor neuron diseases. The total score ranges from 0 to 36, with higher scores indicating greater functional independence. The use of assistive devices and environmental adaptations may influence performance and should be considered during interpretation. 8

The ACTIVLIM is based on the Rasch model, a probabilistic model that places item difficulty and individual ability on a common linear scale, enabling precise and reliable measurements. 7 This model enhances the clinical applicability of the scale by ensuring interval-level properties and robust psychometric characteristics ( Table 2 ).

**Table 2:** Translation of the questions of the Activity Limitations Measure (ACTIVLIM)

	*How difficult are the following activities?*	Qual a sua dificuldade nas seguintes atividades?
1	*Putting on a T-shirt*	Vestir uma camiseta
2	*Washing one's upper body*	Lavar o corpo da cintura para cima
3	*Dressing one's lower body*	Vestir-se da cintura para baixo
4	*Taking a shower*	Tomar banho no chuveiro
5	*Sitting on the toilet*	Sentar-se no vaso sanitário
6	*Taking a bath*	Tomar banho de banheira (ou piscina)
7	*Walking downstairs*	Descer escadas
8	*Stepping out of a bathtub*	Entrar e sair do box no banheiro
9	*Opening a door*	Abrir uma porta
10	*Walking outdoors on leveled ground*	Andar em locais planos fora de casa
11	*Washing one's face*	Lavar o rosto
12	*Hanging up a jacket on a hatstand*	Pendurar um casaco no armário
13	*Wiping one's upper body*	Enxugar o rosto, as mãos e os braços
14	*Walking upstairs*	Subir escadas
15	*Carrying a heavy load*	Carregar um pacote pesado
16	*Getting into a car*	Entrar no carro
17	*Standing for a long time (±10 min)*	Ficar em pé por muito tempo (±10 min)
18	*Walking more than 1 kilometer*	Andar mais de 1 quilômetro
19	*Closing a door*	Fechar uma porta
20	*Hopping on one foot*	Pular num pé só
21	*Putting on a backpack*	Colocar uma mochila
22	*Running*	Correr

To complement the evaluation, the following scales were used:

- Vignos scale: This staging system evaluates lower-limb function in individuals with NMDs. It consists of 10 levels (0–10), considering walking ability, stair use, transitions such as rising from a chair or sitting, and dependence on assistive devices. 12 Higher scores reflect greater functional impairment.
- Brooke Upper Extremity Scale: Comprising 6 levels (1–6), this scale assesses upper-limb function, particularly tasks involving arm elevation and bringing the hands to the mouth. 13 As with the Vignos scale, higher scores indicate more severe limitations.
- Functional Independence Measure (FIM): A widely-used tool to assess disability and care burden, it includes 18 items distributed across domains such as self-care, mobility, bowel and bladder control, communication, and social cognition. Each item is scored from 1 (total dependence) to 7 (complete independence), with the total score ranging from 18 to 126. 14
- Medical Research Council (MRC) scale: Used to grade muscle strength in individual muscle groups on a scale from 0 to 5: 0 = no contraction; 1 = flicker/trace of contraction; 2 = active movement with gravity eliminated; 3 = active movement against gravity; 4 = active movement against some resistance; and 5 = normal strength. A strength index was also calculated using the formula MRC index = (total score × 100)/(number of muscles tested × 5), yielding a percentage that reflects global muscle strength. 15

### Statistical analysis

Statistical analyses were conducted using the Statistica software, version 13.0 (StatSoft Inc.). The psychometric properties of the Brazilian version of the ACTIVLIM were examined through analyses of reliability, internal consistency, construct validity (external consistency), and responsiveness.

The inclusion of 60 participants in the assessment of psychometric properties was based on established guidelines for instrument validation. 9 To assess test-retest reliability, these participants were evaluated twice by the same rater, with a 1-month interval between sessions. The same subgroup was also assessed at baseline by a second, blinded rater to test interrater reliability. Intraclass correlation coefficients (ICCs) were calculated for the intra- and interexaminer reliability, with values ≥ 0.80 considered indicative of high reliability. 16 After reliability was confirmed through these analyses, the test was applied to the larger group of 268 patients, ensuring that the psychometric properties of the instrument were robust and could be generalized. 17

Internal consistency was evaluated using the Cronbach's alpha. This index reflects the homogeneity of the items, indicating whether they assess the same construct. Higher interitem correlations result in increased alpha values, with those ≥ 0.70 generally considered acceptable. 16

Construct validity (external consistency) was tested using Spearman's rank correlation coefficients, adjusted for age, to examine associations involving the ACTIVLIM and established functional measures: the Vignos scale, the Brooke scale, and the FIM. To control multiple comparisons, the Bonferroni correction was applied. Bonferroni correction consists of adjusting the significance threshold to p < 0.017(as a result of 0.05 / 3, because there were three comparisons). Additionally, the relationship between the ACTIVLIM and muscle strength (measured by the MRC scale) was also assessed. Correlation coefficients ≥ 0.80 were interpreted as strong. 18

The responsiveness of the Brazilian version of the ACTIVLIM was examined using linear regression analysis, in which the coefficient of determination (R ^2^ ) was used to estimate the proportion of variance in ACTIVLIM scores explained by independent variables (such as functional staging and muscle strength). The values of R ^2^ range from 0 to 1, with higher values indicating a better model fit and greater explanatory power, relative to the sample size and degrees of freedom (dfs).

## RESULTS

For the intra- and interrater reliability analysis, data from 60 participants evaluated by 2 physiotherapists, with a 1-month interval between assessments, were analyzed. Values of ICC of 0.95 (intrarater) and 0.90 (interrater) were obtained, indicating excellent reliability.

Regarding internal consistency, the Cronbach's alpha for the total ACTIVLIM score was of 0.94, classified as high. The alpha values for individual items also demonstrated high homogeneity, ranging from 0.94 to 0.95.

For construct validity (external consistency), Spearman's correlation coefficients (adjusted for age) were calculated. As multiple comparisons were made, a Bonferroni correction adjusted the significance threshold to *p* < 0.006 (0.050/8). Strong and statistically significant correlations were observed regarding the ACTIVLIM and the Vignos scale ( *r* = −0.907), the Brooke Scale ( *r* = −0.908), and the FIM ( *r* = 0.864), with *p* < 0.001 for all comparisons.

**Figure 1:**
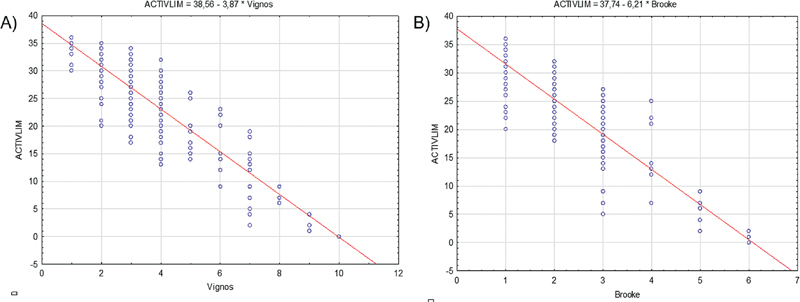
( **A** ) Relationship between patients' activity measures and the Vignos scale. ( **B** ) Relationship between patients' activity measurse and the Brooke scale.

A strong negative correlation was found involving the ACTIVLIM scores and disease staging using the Vignos and Brooke scales ( Figure 1 ). Higher ACTIVLIM scores reflected greater functional independence, while lower scores were associated with increased disease severity and dependence in daily living activities.

**Figure 2:**
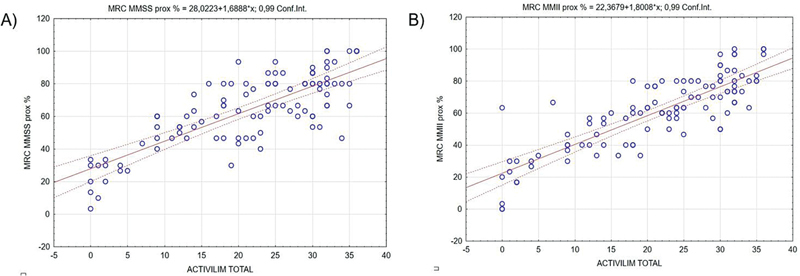
( **A** ) Correlation and relationship between the Activity Limitation Measure (ACTIVLIM) and proximal muscle strength of the upper limbs (according to the Medical Research Council [MRC] scale). ( **B** ) Correlation and relationship between the ACTIVLIM and proximal muscle strength of the lower limbs.

Figure 2 illustrates the correlations regarding muscle strength assessed by the MRC scale and ACTIVLIM scores. A strong positive correlation was found involving proximal muscle strength of the upper ( *r* = 0.748) and lower limbs ( *r* = 0.793) and the ACTIVLIM scores ( *p* < 0.001 for both).

Other muscle strength domains showed moderate correlations with the ACTIVLIM scores, such as trunk muscle strength ( *r* = 0.602), distal upper limb strength ( *r* = 0.512), and distal lower limb strength ( *r* = 0.594), with *p* < 0.001 for all comparisons.

For a clearer understanding of group characteristics, the participants were categorized into subgroups according to diagnosis: SMA, congenital muscular dystrophy (CMD), Duchenne/Becker muscular dystrophy (DMD/BMD), facioscapulohumeral dystrophy (FSH), Steinert's myotonic dystrophy, congenital myopathy, inflammatory myopathy, mitochondrial myopathy, and other NMDs (such as, neuropathies, myasthenias, amyotrophic lateral sclerosis, Emery-Dreifuss dystrophy, and oculopharyngeal dystrophy).

We investigated the correlation between the total ACTIVLIM scores and muscle strength, assessed by the MRC scale. Using simple linear regression, we identified the variables that best explained the variance in ACTIVLIM scores:

- Vignos scale: R ^2^ = 0.8236; estimate = −1.0267; df = 1;
- Brooke scale: R ^2^ = 0.8132; estimate = −0.9981; df = 1;
- Proximal lower limb muscle strength: R ^2^ = 0.6480; estimate = 1.0049; df = 1; and
- Proximal upper limb muscle strength: R ^2^ = 0.5805; estimate = 0.9605; df = 1.

**Figure 3:**
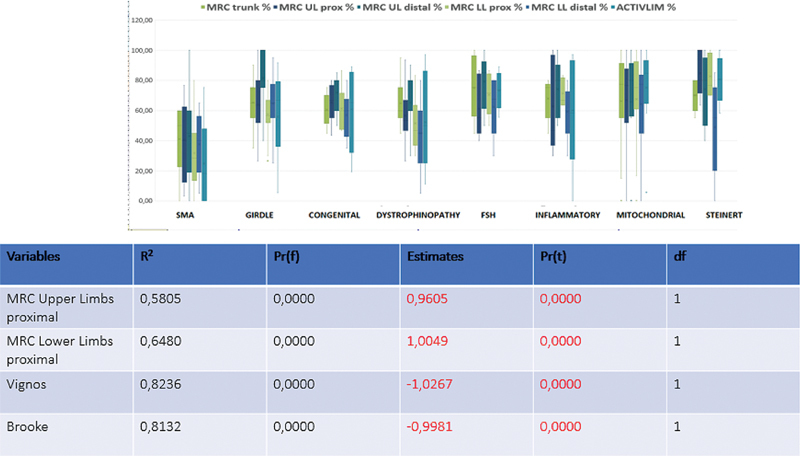
Correlation of the MRC scale (global muscle strength) and the ACTIVLIM, with the participants divided by specific diagnosis and linear regression.

These findings demonstrate that the functional staging scales and proximal muscle strength are strong predictors of functional independence, as measured by the ACTIVLIM ( Figure 3 ).

## DISCUSSION

In the current study, we translated, cross-culturally adapted, and validated the Brazilian Portuguese version of the ACTIVLIM questionnaire. In line with the ICF, the evaluation extended beyond body structure and functions, such as muscle strength, incorporating broader dimensions such as activity, participation, and contextual factors. Functional measures are increasingly preferred in monitoring NMD progression, 19 and the ACTIVLIM aligns with this perspective by assessing limitations in activities of daily living.

The translation process followed the guidelines proposed by Beaton et al., 9 which proved to be effective for developing the Brazilian Portuguese version of the ACTIVLIM. Subsequent analyses demonstrated excellent reliability: ICCs of 0.95 (intrarater) and 0.90 (interrater), similar to the original study by Vandervelde et al. 7 (who found values of 0.98 and 0.93 respectively). Comparable results were observed in the Spanish validation by Pagola et al., 19 in which 135 adults with myopathies yielded an ICC of 0.96. These findings confirm the high reproducibility and reliability of the Brazilian Portuguese version.

Internal consistency was also high, with a Cronbach's alpha of 0.94, indicating strong homogeneity among the questionnaire items. In terms of construct validity, our correlations involving the ACTIVLIM and the Vignos scale (r = −0.907), the Brooke scale (r = −0.908), and the FIM (r = 0.864) were similar or stronger than those reported in the original questionnaire and in the Spanish validation. 7 19 These inverse relationships reflect that greater ACTIVLIM scores indicate better functional independence, while higher scores on the Vignos or Brooke scales denote increased disability. Thus, the ACTIVLIM complements other tools by incorporating the patient's perception, which is critical for clinical reasoning and to identify tasks and muscle synergies to prioritize during therapy.

In the analysis of the ACTIVLIM items, common difficulties involved lower-limb activities such as climbing up and down stairs, walking outdoors, prolonged standing, and walking long distances. These challenges align with higher severity levels in the Vignos scale. Difficulties with upper-limb activities, such as putting on a T-shirt, washing the upper body, or reaching overhead, correlate with the Brooke scale scores, which reflect upper-limb mobility and strength.

Most participants reported impairments in lower-limb function. Accordingly, the linear regression identified proximal lower-limb strength and the Vignos and Brooke scales as the best predictors of ACTIVLIM scores, supporting the scale's relevance in the clinical monitoring of NMDs.

Although the FIM and ACTIVLIM demonstrated a strong correlation, they serve different purposes. The FIM measures overall functional independence and participation in social contexts, 14 while the ACTIVLIM focuses on self-perceived difficulty in performing daily tasks. Notably, the ACTIVLIM allows for compensatory strategies but does not account for assistance from others, 20 which is an important distinction in interpreting patient performance in NMDs, in which compensatory patterns are common. Clinical observations often reveal altered postures and gait, such as equinus foot, widened stance, pelvic tilt, lumbar hyperlordosis, and internal rotation of the shoulders, 21 reflecting strategies to overcome antigravity muscle weakness.

Previous studies 13 have primarily explored the relationship between muscle strength and motor function in DMD. In the current study, this relationship extended to other NMDs, with strong correlations involving the ACTIVLIM and proximal upper- and lower-limb strength. As reported by Vandervelde et al., 22 shoulder and elbow flexors are essential for tasks such as washing the face or putting on a T-shirt, while hip and knee flexors contribute to climbing stairs or stepping out of a bathtub.

Manual muscle testing (MMT) and dynamometry remain essential for the clinical evaluation. While dynamometry quantifies peak force, MMT provides qualitative insights on movement patterns and gravity resistance. When applied by experienced clinicians, MMT demonstrates good reproducibility and is especially valuable in clinical reasoning for individuals with NMDs. 23 24 Functional assessments such as the ACTIVLIM add contextual depth to strength testing by linking impairments with daily challenges.

Nunes et al. 23 also demonstrated strong correlations between proximal and distal muscle strength in the Motor Function Measure (MFM), particularly in tasks involving trunk stability—critical for transfers and mobility. Our findings reinforce this: proximal strength in the upper and lower limbs correlated strongly with the ACTIVLIM scores (r = 0.748 and 0.793 respectively), supporting the importance of trunk and limb stability for complex tasks such as climbing stairs or hanging clothes. 6

Bakker et al. 25 found that muscle strength, as measured by the MRC scale, could predict gait loss in DMD. Hip extensor strength ≤ 2 and dorsiflexor strength ≤ 4 were associated with increased risk. Combining the MRC and the ACTIVLIM may enhance clinical monitoring in NMDs. Future studies should investigate the responsiveness of these instruments in combination.

Finally, incorporating the patient's perspective—as captured by the ACTIVLIM—and adopting patient- and family-centered care practices broadens the focus of rehabilitation. 23 26 There is evidence 27 28 that engaging patients in goal-setting improves therapy adherence, functional outcomes, pain management, range of motion, and strength. Thus, the ACTIVLIM is a valuable tool to integrate patient-reported outcomes into clinical decision-making for individuals with NMDs.

In conclusion, the Brazilian Portuguese version of the ACTIVLIM demonstrated excellent reliability, internal consistency, and strong construct validity through significant correlations with functional staging, independence measures, and muscle strength. By assessing patients' self-perception of activity limitations–particularly in self-care and mobility—the ACTIVLIM offers a meaningful and sensitive tool to evaluate the functional impact of NMDs.

Aligned with the ICF framework and centered on patient-reported outcomes, the ACTIVLIM complements clinical assessments and supports individualized rehabilitation planning. Its use is recommended in the routine clinical practice and research in Brazil, and future studies should explore its responsiveness to interventions and longitudinal applicability in diverse care settings.
